# Show us your ticks: a survey of ticks infesting dogs and cats across the USA

**DOI:** 10.1186/s13071-019-3847-3

**Published:** 2019-12-19

**Authors:** Meriam N. Saleh, Kellee D. Sundstrom, Kathryn T. Duncan, Michelle M. Ientile, Julia Jordy, Parna Ghosh, Susan E. Little

**Affiliations:** Department of Veterinary Pathobiology, Center for Veterinary Health Sciences, Oklahoma State University, Stillwater, OK 74078 USA

**Keywords:** *Amblyomma*, Attachment site, Cat, *Dermacentor*, Dog, Ixodidae, *Ixodes*, *Rhipicephalus*, Tick

## Abstract

**Background:**

A variety of tick species infest dogs and cats in North America. Although most of these species also readily feed on people, national data regarding the species and abundance of ticks on dogs and cats are lacking. Here we report a large-scale study of ticks from dogs and cats in the USA over a 12-month period.

**Methods:**

Tick submissions were invited from veterinary practices in all 50 states. Ticks were submitted with information about the pet and the attachment sites of each tick marked on a biopsy chart. Upon receipt, ticks were identified to species and stage using morphologic keys; when necessary, species identification was confirmed molecularly.

**Results:**

From February 2018 through January 2019, 10,978 ticks were submitted from 1494 dogs and 336 cats in 49 states and ticks were collected in every month. Dog and cat infestation intensities ranged from 1 to 4765 and from 1 to 38 (median = 1, mean = 6.7 and 2.6), respectively. Dogs were primarily infested with *Dermacentor variabilis* (532/1494; 35.6%), *Ixodes scapularis* (409/1494; 27.4%), *Amblyomma americanum* (345/1494; 23.1%) and *Rhipicephalus sanguineus* (172/1494; 11.5%). Cats were primarily infested with *I. scapularis* (156/336; 46.4%), *A. americanum* (99/336; 29.5%) and *D. variabilis* (60/336; 17.9%). Other submitted ticks included *A. maculatum*, *Haemaphysalis longicornis*, *Otobius megnini*, and less common *Dermacentor* spp. and *Ixodes* spp. Co-infestations were documented in 93 dogs and 14 cats. Reported attachment sites of common tick species differed. In dogs, *A. americanum* was most commonly attached to the abdomen, axillary, and inguinal regions; *D. variabilis* and *I. scapularis* to the head, neck, and back; and *R. sanguineus* to the head, neck, abdomen, legs, and feet. In cats, *I. scapularis* was most commonly attached to the head and *A. americanum* was most commonly attached to the tail and perianal region.

**Conclusions:**

These data confirm that dogs and cats in the USA are at risk of tick infestation throughout the year and that tick species present in the region have apparent attachment site preferences.
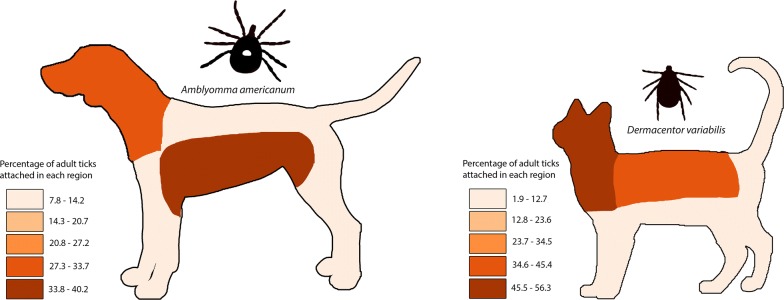

## Background

Ticks are common ectoparasites of significant medical and veterinary importance worldwide. Several different tick species, most of which transmit zoonotic and veterinary pathogens, are known to feed on domestic dogs and cats (Tables 1, 2). In the USA, common species include *Amblyomma americanum*, *A. maculatum*, *Dermacentor variabilis*, *Ixodes scapularis*, *I. pacificus* and *Rhipicephalus sanguineus* [1], but recent, comprehensive surveys from this region documenting the species and occurrence of ticks on pets and particularly on dogs, are lacking. The close association between people and pets, along with the shared disease risk ticks pose, has fostered recent interest in large-scale surveys of ticks from companion animals [2, 3]. A review of medical records from 2002– 2004 reported that ticks were found on 29,662/2,275,048 (1.3%) dogs in 40 states, but information on species or stage was not available [4]. A “citizen-science” survey detailed tick infestations on people and animals across the USA, but did not report which tick species were found on dogs and cats or in the different geographical regions [5].

**Table 1:** Representative published reports of ticks recovered from dogs

Study population (*N*; %)	Ticks identified (*n*; tick stages^a^)	Location	References
Public Health England’s passive Tick Surveillance Scheme (TSS) (1580; nr)	*Ixodes ricinus* (2104; F/M/N/L)	UK	[42]
	*Ixodes hexagonus* (943; F/M/N/L)		
	*Haemaphysalis punctata* (164; F/M/L)		
	*Dermacentor reticulatus* (47; F/M)		
	*Ixodes canisuga* (18; F/N/L)		
Pet dogs presented to veterinarians (1383/3026; 45.7%)	*Rhipicephalus sanguineus* (*s.l.*) (1822; F/M/N/L)	Italy	[39]
	*I. ricinus* (468; F/M/N/L)		
	*I. hexagonus* (83; F/M/N)		
	*Dermacentor marginatus* (5; F/M)		
	*Rhipicephalus bursa* (11; F/M/N)		
	*D. reticulatus* (7; F/M)		
	*H. punctata* (4; F/N)		
	*Ixodes arboricola* (32; L)		
	*I. canisuga* (4; F/N)		
	*Ixodes gibbosus* (2; F)		
	*Ixodes festai* (1; F)		
Pet dogs presented to veterinarians (nr)	*I. ricinus* (95; A)	Belgium, France, Hungary, Italy	[43]
	*R. sanguineus* (*s.l.*) (74; A)		
	*D. reticulatus* (43; A)		
	*I. hexagonus* (12; A)		
Pet dogs presented to veterinarians (562; nr)	*R. sanguineus* (*sensu lato*) (1058)	China	[44]
	*Haemaphysalis longicornis* (286)		
	*Rhipicephalus haemaphysaloides* (195)		
	11 too damaged for identification (A/N/L; species nos. for each stage not specified)		
Pet dogs presented to veterinarians (180; nr)	*R. sanguineus* (*s.l.*) (1242)	USA (Florida)	[9]
	*Amblyomma americanum* (36)		
	*Ixodes scapularis* (24)		
	*Dermacentor variabilis* (10)		
	*Amblyomma maculatum* (4)		
Pet dogs presented to veterinarians and individual submissions (643; nr)	*R. sanguineus* (*s.l.*) (3069; F/M/N/L)	Australia	[33]
	*Ixodes holocyclus* (770; F/M/N)		
	*H. longicornis* (213; F/N/L)		
	*Ixodes tasmania* (90; F/M/N/L)		
	*Ixodes cornuatus* (15; F/N)		
	*Bothriocroton* sp. (14; F/M/N/L)		
	*Amblyomma triguttatum triguttatum* (10; F/N)		
	*Haemaphysalis bancrofti* (5; F/N)		
	*Ixodes myrmecobii* (4; F)		
	*Rhipicephalus australis* (1; N)		
Pet dogs presented to veterinarians (1162; nr)	*H. longicornis* (2633; F/M/N/L)	Japan	[34]
	*R. sanguineus* (*s.l.*) (882; F/M/N/L)		
	*Haemaphysalis flava* (316; F/M/N/L)		
	*Ixodes ovatus* (182; F/M/N)		
	*Haemaphysalis hystricis* (33; F/M/N)		
	*Haemaphysalis megaspinosa* (30; F/N/L)		
	*Ixodes nipponensis* (30; F/M/N)		
	*Ixodes persulcatus* (27; F/M)		
	*Amblyomma testudinarium* (22; F/N/L)		
	*Haemaphysalis campanulata* (19; F/M)		
	*Haemaphysalis japonica* (11; F/M/N)		
	*Haemaphysalis* spp. (17; F/N/L)		
	*Ixodes* spp. (2; F)		
	*Haemaphysalis formosensis* (1; N)		
	*Haemaphysalis ias* (1; F)		
	Unidentified (31; F/M/N/L)		
Pet dogs during rabies vaccination campaign and pet dogs at selected. Home (154/413; 37.3%)	*R. sanguineus* (*s.l.*) (674; A/N)	Brazil	[45]
	*Amblyomma* sp. (146; N/L)		
	*Amblyomma cajennense* (6; A)		
	*Amblyomma ovale* (7; A)		
	*Rhipicephalus* (*Boophilus*) *microplus* (2; N)		
Pet and shelter dogs at selected locations (nr)	*I. scapularis* (1147; M/F)	USA (Georgia)	[7]
	*D. variabilis* (628; M/F/N)		
	*R. sanguineus* (274; F/M/N)		
	*A. maculatum* (218; F/M/N)		
	*A. americanum* (111; F/M/N)		
	*Amblyomma tuberculatum* (72; L)		
	*Ixodes affinis* (14; M/F)		
	*Haemaphysalis leporispalustris* (1; F)		
	*Ixodes cookei* (1; F)		
Pet dogs at selected homes (870; nr)	*A. americanum* (23676; F/M/N/L)	USA (Oklahoma, Arkansas)	[6]
	*R. sanguineus* (46652; F/M/N/L)		
	*I. scapularis* (965; M/F)		
	*D. variabilis* (717; M/F)		
	*A. maculatum* (10; F/M/N)		
	*I. cookei* (5; F)		

^a^Provided when specified in reference

*Abbreviations*: F, female; M, male; N, nymph; L, larva; A, adult; N, number infested; n, number of ticks; nr, not reported

Several detailed reports of ticks on pets in limited geographical areas of the USA are available (Tables 1, 2) [2, 6–14], but none are national in scope. Compiling current, comprehensive data about ticks infesting dogs and cats in the USA is time- and resource-intensive but critically important for both veterinary and human health [15]. Improved knowledge of the tick species that pets encounter across the USA can provide valuable information about the geographical distribution of ticks throughout the country and thus the risk posed to humans that share the same environment. Because tick removal was documented at veterinary practices in the present study, we were also able to gain insight into attachment site preferences. The purpose of the present study was to determine the species and stages of ticks infesting dogs and cats throughout the USA and determine tick-host attachment site preferences.

**Table 2:** Representative published reports of ticks recovered from cats

Study population (*N*; %)	Ticks identified (*n*; tick stages^a^)	Location	References
Public Health England’s Tick Surveillance Scheme (TSS) (568; nr)	*Ixodes hexagonus* (918; F/M/N/L)	UK	[42]
	*Ixodes ricinus* (384; F/M/N)		
	*Ixodes canisuga* (3; F/N)		
	*Ixodes ventalloi* (3; F)		
	*Ixodes frontalis* (1; F)		
	*Haemaphysalis punctata* (1; F)		
Pet cats presented to veterinarians (332; nr)	*Ixodes scapularis* (423; F/M/N/L)	USA	[2]
	*Amblyomma americanum* (226; F/M/N/L)		
	*Dermacentor variabilis* (131; F/M/N)		
	*Ixodes pacificus* (11)		
	*Ixodes banksi* (1)		
	*Dermacentor occidentalis* (1)		
	*Amblyomma maculatum* (1)		
	*Otobius megnini* (1)		
	*Rhipicephalus sanguineus* (*s.l.*) (1)		
Pet cats presented to veterinarians (nr)	*I. ricinus* (152; A)	Germany, France, Hungary and Italy	[43]
	*R. sanguineus* (*s.l.*) (42; A)		
	*Dermacentor reticulatus* (16; A)		
	*I. hexagonus* (14; A/N)		
Pet cats presented to veterinarians and individual submissions (152; nr)	*Ixodes holocyclus* (185; F/M/N)	Australia	[33]
	*Ixodes tasmania* (39; F/N/L)		
	*Haemaphysalis bancrofti* (1; F)		
	*Ixodes cornuatus* (1; F)		
	*Ixodes hirsti* (1; F)		
	*Ixodes myrmecobii* (1; F)		
	*R. sanguineus* (*s.l.*) (1; F)		
Pet cats presented to veterinarians and free-roaming cats presented to spay/neuter program (37/308; 12%)	*I. ventalloi* (62; F/M)	Italy (Sicily, Calabria)	[46]
	*I. ricinus* (20; F/M)		
	*Ixodes* spp. (5; F)		
	*R. sanguineus* (*s.l.*) (28; F/M)		
	*Rhipicephalus pusillus* (17; M)		
Pet cats presented to veterinarians (136; nr)	*Haemaphysalis longicornis* (106; F/M/N/L)	Japan	[34]
	*Amblyomma testudinarium* (80; F/N/L)		
	*Ixodes ovatus* (55; F/M/N)		
	*Haemaphysalis flava* (18; F/N/L)		
	*Haemaphysalis hystricis* (12; N)		
	*Ixodes nipponensis* (10; F/N)		
	*Ixodes persulcatus* (6; F)		
	*Haemaphysalis japonica* (2; N)		
	*R. sanguineus* (*s.l.*) (2; F)		
	*Haemaphysalis megaspinosa* (1; L)		
	*Ixodes granulatus* (1; F)		

^a^Provided when specified in reference

*Abbreviations*: F, female; M, male; N, nymph; L, larva; A, adult; N, number infested; n, number of ticks; nr, not reported

## Methods

### Tick collections

Ticks submissions were invited from 190 enrolled veterinary practices in all 50 states to ensure broad geographical representation and were supplemented by submissions from other veterinary practices interested in supporting the study. Each practice was provided with instructions and submission kits containing forceps, tick containers, prepaid mailing envelopes and submission forms. Instructions for tick submissions were also made available on a study website [16]. Ticks identified on a dog or cat were removed and placed in a hard-plastic container with a tightly fitting lid which was then sealed in a plastic bag with a completed submission form and shipped to Oklahoma State University; occasionally ticks were submitted in serum tubes or similar hard, tightly sealed containers. The submission form collected information on removal date of tick; age, weight, sex, spay/neuter status and breed of pet; owner reported estimate of percent time the pet spent outside; and a diagram to indicate the tick attachment location(s) on the dog or cat. When multiple ticks were present we requested that all ticks be collected and submitted.

### Tick identification

Ticks were immediately examined upon receipt, the stage (female, male, nymph, larva) of each tick recorded and tick genus and species determined using standard keys [17–23]. After identification, specimens were held in 70% ethanol at − 20 °C. An e-mail was sent to the submitting veterinarian with the initial morphologic identification and a list of pathogens that species/stage is known to transmit, if any. When damage to the specimen precluded identification by morphology, or if the species identification was unusual or uncertain due to morphologic similarity between congeners, ticks were bisected to retain anterior morphologic features and nucleic acid extracted from the posterior half with a commercial kit (Illustra GenomicPrep Kit, GE Healthcare, Marlborough, MA, USA) for molecular identification. Briefly, PCR amplification and direct sequencing of a *16S* rRNA gene fragment [24, 25] was utilized for *Ixodes*, *Haemaphysalis* and *Amblyomma*, a *cox*1 gene fragment [26] was also utilized for *Ixodes* and *Haemaphysalis* and an ITS2 gene fragment [27] was used for *Dermacentor.* Amplicons were visualized in GelRed-stained (Biotium, Inc., Freemont, CA, USA) agarose gels to confirm expected size and purified using a commercial kit according to manufacturer’s instructions (Wizard® SV Gel and PCR Clean-Up System, Promega, Madison, WI, USA). Sequence analysis and alignment were performed using MacVector software (MacVector, Inc., Cary, NC, USA) and were compared with available sequences using the nucleotide Basic Local Alignment Search Tool (BLASTn, National Center for Biotechnology Information, Bethesda, MD, USA). Sequence identity was confirmed *via* visual inspection of the chromatogram and identity to available sequences. Anterior halves of bisected ticks were retained in 70% ethanol at − 20 °C.

### Data management and quality assurance

Tick identification including number of ticks submitted, species and stage was recorded in a log along with the patient information. All data were entered into spreadsheets (Microsoft Excel version 16.16.8). Prior to summary and statistical analyses, quality assurance was performed by reviewing both individual identifications and data entry. Attachment site data were recorded from marked biopsy charts on original submission cards. Regions of the body were divided into 5 areas for analysis: head, ears and neck; abdomen, axillary and inguinal; legs and feet; back; and tail and perianal region. Attachment site was only assessed for dogs and cats infested with a single species of adult tick.

### Statistical analyses

Statistical analyses were performed using JMP (Version 12. SAS Institute Inc., Cary, NC, 1989–2019). Confidence intervals (CI 95%) were calculated for average reported weight and age. Chi-square tests, with significance levels below α = 0.05, were performed to evaluate differences in sex and altered status of dogs and cats with ticks compared to that reported from the general pet population in the USA and to evaluate differences in tick attachment site on dogs and cats among the most common tick species received. Percent ranked quintiles were established for tick attachment site data to depict attachment site preferences graphically.

## Results

### Dogs with ticks

A total of 263 veterinary practices in 49 states (all but North Dakota, USA) submitted 10,087 ticks from 1494 dogs (Table 3). Practices that submitted ticks were located in the Northeast (*n* = 42), South (*n* = 100), Midwest (*n* = 96) and West (*n* = 25). An average of 6.7 ticks (median 1) were submitted from each dog and infestation intensity ranged between 1–4765, with 82 (5.5%) dogs infested with 10 or more ticks. Reported weight of dogs with ticks varied from 0.16 to 90.7 kg (mean 20.1 kg; 95% CI: 19.5–20.8 kg) and reported age ranged from 40 days to 19 years (mean 4.8 years; 95% CI: 4.5–8.2 years). Estimated percent time outside as reported by owner was categorized as < 1% (7/1042; 0.7%), 1–30% (509/1042; 48.8%), 31–70% (290/1042; 27.8%) and > 70% (236/1042; 22.6%); for 452 dogs, an estimate of time spent outside was not provided. Of the dogs for which sex and altered status were provided 719/1438 (50.0%) were male and 720/1438 (50.0%) were female, which is not significantly different than the estimates of males and females for the general pet population of dogs (*χ*^2^ = 1.970, *df* = 1, *P* = 0.1595) [28]; 441/718 (61.4%) of males were neutered, which is not significantly different than the estimates for the general pet population of dogs (*χ*^2^ = 0.02, *df* = 1, *P* = 0.9690) [28] and 454/719 (63.1%) of females were spayed which is significantly different than the estimates for the general population where 67.4% of females were spayed (*χ*^2^ = 6.02, *df* = 1, *P* = 0.0142) [28].

**Table 3:** Ticks collected from domestic dogs in the USA by species, stage and month of collection

Species	Stage	Total	Jan	Feb	Mar	Apr	May	Jun	Jul	Aug	Sep	Oct	Nov	Dec
*Rhipicephalus sanguineus*	F	486	14	0	13	23	24	65	209	96	17	13	11	1
	M	617	45	0	27	30	29	65	227	160	12	20	2	0
	N	1120	0	0	0	1	0	40	1016	30	32	1	0	0
	L	4029	0	0	0	0	0	5	4005	19	0	0	0	0
*Amblyomma americanum*	F	514	0	0	12	54	163	172	35	69	8	0	0	1
	M	292	4	0	14	50	90	102	17	11	4	0	0	0
	N	363	0	0	2	12	44	129	47	75	53	1	0	0
	L	762	4	0	0	0	1	0	242	84	418	13	0	0
*Dermacentor variabilis*	F	631	0	0	0	21	198	172	142	46	45	4	3	0
	M	392	0	0	0	25	128	105	74	20	36	3	1	0
	N	2	0	0	0	0	0	1	1	0	0	0	0	0
	L	0	0	0	0	0	0	0	0	0	0	0	0	0
*Ixodes scapularis*	F	489	37	6	8	34	34	14	5	0	4	158	151	38
	M	84	6	3	2	1	2	0	0	0	1	37	21	11
	N	3	0	0	0	0	0	1	0	1	0	1	0	0
	L	0	0	0	0	0	0	0	0	0	0	0	0	0
Other	F	128	7	13	6	2	4	6	17	31	21	5	12	4
	M	118	2	1	0	4	3	11	19	41	29	5	3	0
	N	53	0	0	26	0	1	0	1	10	3	11	0	1
	L	4	0	0	0	0	0	0	0	4	0	0	0	0
Total		10,087	119	23	110	257	721	888	6057	697	683	272	204	56

*Note*: Other submitted ticks included *A. maculatum*, *I. pacificus*, *O. megnini*, *I. affinis*, *I. cookei*, *I. angustus*, *Ixodes* sp., *H. longicornis*, *D. albipictus* and *D. andersoni*

*Abbreviations*: F, female; M, male; N, nymph; L, larva

Of the 1494 dogs with ticks, *D. variabilis* was present on 35.6% (532/1494), *Ixodes scapularis* on 27.4% (409/1494), *A. americanum* on 23.1% (345/1494) and *R. sanguineus* on 11.4% (174/1494). A smaller number of dogs were infested with *A. maculatum* (98/1494; 6.6%), *I. pacificus* (22/1494; 1.5%), or *Otobius megnini* (6/1494; 0.4%). A few dogs were found to be infested with *I. angustus* (*n* = 5), *I. cookei* (*n* = 4), *I. affinis* (*n* = 4), *Ixodes* sp. (*n* = 1), *D. albipictus* (*n* = 2), or *D. andersoni* (*n* = 1). Co-infestations with more than one tick species were documented on 93 dogs.

### Cats with ticks

A total of 109 veterinary practices in 39 states submitted 891 ticks from 336 cats (Table 4). These practices were located in the Northeast (*n* = 25), South (*n* = 40), Midwest (*n* = 30) and West (*n* = 14). An average of 2.6 ticks (median 1) were submitted from each cat and infestation intensity ranged from 1 to 38, with 16 (4.8%) cats infested by 10 or more ticks. Reported weight of cats with ticks varied from 0.18 to 13.5 kg (mean 4.4 kg; 95% CI: 3.9–8.6 kg) and reported age ranged from 18 days to 18 years (mean 4.4 years; 95% CI: 3.9–8.6 years). Estimated percent time outside as reported by owner was categorized as none (12/283; 4.2%), 0.5–30% (36/283; 12.7%), 31–70% (75/283; 26.5%) and > 70% (160/283; 56.5%); for 53 cats an estimate of time spent outside was not provided. Of the cats for which sex and altered status were provided 194/331 (58.6%) were male and 137/331 (41.4%) were female; 130/194 (67.0%) of males were neutered and 90/137 (65.7%) of females were spayed which is significantly different than the estimates for the general pet population of cats where only 49.6% of pet cats were male and 50.4% were female (*χ*^2^ = 10.60, *df* = 1, *P* = 0.0011); and 83% of males and 81% of females were altered (*χ*^2^ = 36.87, *df* = 1, *P* < 0.0001; *χ*^2^ = 22.34, *df* = 1, *P* < 0.0001) [28].

**Table 4:** Ticks collected from domestic cats in the USA by species, stage and month of collection

Species	Stage	Total	Jan	Feb	Mar	Apr	May	Jun	Jul	Aug	Sep	Oct	Nov	Dec
*Amblyomma americanum*	F	93	0	0	2	6	45	33	6	0	1	0	0	0
	M	32	0	0	1	2	15	11	0	1	1	0	1	0
	N	92	0	0	2	9	27	20	5	22	6	1	0	0
	L	126	0	0	0	0	0	7	27	41	39	12	0	0
*Dermacentor variabilis*	F	48	0	0	1	1	20	13	7	1	1	0	4	0
	M	41	0	0	0	4	13	7	8	1	0	0	8	0
	N	1	0	0	0	0	0	0	0	1	0	0	0	0
	L	32	0	0	0	0	0	0	0	0	32	0	0	0
*Ixodes scapularis*	F	225	8	0	4	2	12	4	1	0	3	110	53	28
	M	41	2	0	0	0	1	2	0	0	0	28	5	3
	N	19	0	0	0	1	7	8	1	0	2	0	0	0
	L	2	0	0	0	0	0	2	0	0	0	0	0	0
Other	F	20	0	2	0	0	1	5	8	2	1	0	1	0
	M	4	1	0	0	0	0	0	3	0	0	0	0	0
	N	115	22	0	0	0	0	9	3	29	18	13	4	17
	L	0	0	0	0	0	0	0	0	0	0	0	0	0
Total		891	33	2	10	25	141	121	69	98	104	164	76	48

*Note*: Other submitted ticks included *O. megnini*, *R. sanguineus* (*s.l.*), *A. maculatum*, *D. albipictus*, *I. affinis*, *I. angustus*, *I. pacificus*, *H. longicornis* and *D. andersoni*

*Abbreviations*: F, female; M, male; N, nymph; L, larva

Of the 336 cats with ticks, *I. scapularis* was present on 46.4% (156/336), *A. americanum* on 29.5% (99/336) and *D. variabilis* on 17.9% (60/336). A smaller number of cats were infested with *O. megnini* (13/336; 3.9%), *R. sanguineus* (5/336; 1.5%), *A. maculatum* (5/336; 1.5%), or *D. albipictus* (4/336; 1.2%). A few cats were found to be infested with *I. pacificus* (*n* = 3), *I. affinis* (*n* = 3), *I. angustus* (*n* = 1), *D. andersoni* (*n* = 2), or *H. longicornis* (*n* = 1). Co-infestations with more than one tick species were documented on 14 cats.

### Tick species and stages identified

In dogs, 14 tick species were identified (Table 3). The majority of ticks submitted from dogs were *R. sanguineus* (*sensu lato*) (6252/10,087; 62.0%), *A. americanum* (1931/10,087; 19.1%), *D. variabilis* (1025/10,087; 10.2%) and *I. scapularis* (576/10,087; 5.7%) (Table 3). A number of other tick species were submitted including *A. maculatum* (*n* = 188), *O. megnini* (*n* = 35), *I. pacificus* (*n* = 34), *I. affinis* (*n* = 16), *I. cookei* (*n* = 15), *I. angustus* (*n* = 5), *Ixodes* sp. (*n* = 4), *H. longicornis* (*n* = 3), *D. albipictus* (*n* = 2) and *D. andersoni* (*n* = 1).

In cats, 12 tick species were identified (Table 4). The majority of ticks submitted were *A. americanum* (343/891; 38.5%), *I. scapularis* (287/891; 32.2%) and *D. variabilis* (122/891; 13.7%) (Table 4). Other submitted tick species included *O. megnini* (*n* = 74), *A. maculatum* (*n* = 32), *R. sanguineus* (*sensu lato*) (*n* = 14), *D. albipictus* (*n* = 6), *I. affinis* (*n* = 5), *I. pacificus* (*n* = 3), *H. longicornis* (*n* = 2), *D. andersoni* (*n* = 2) and *I. angustus* (*n* = 1). The majority of submitted ticks were larvae (4985/10,978; 45.4%), followed by adult females 2635/10,978; 24.0%), nymphs (1737/10,978; 15.8%) and adult males (1621/10,978; 14.8%). Adult females were the predominant stage of *D. variabilis* (679/1147; 59.2%) and *I. scapularis* (714/863; 82.7%) submitted, while larvae were the majority of *R. sanguineus* (4029/6266; 64.3%) and *A. americanum* (888/2274; 39.1%) submitted. Ticks were submitted in every month of the year, with the highest number of ticks recovered in July (6126/10,978; 55.8%) and primarily consisting of *R. sanguineus* (5467/6126; 89.2%) (Tables 3, 4).

### Site of tick attachment

**Fig. 1:**
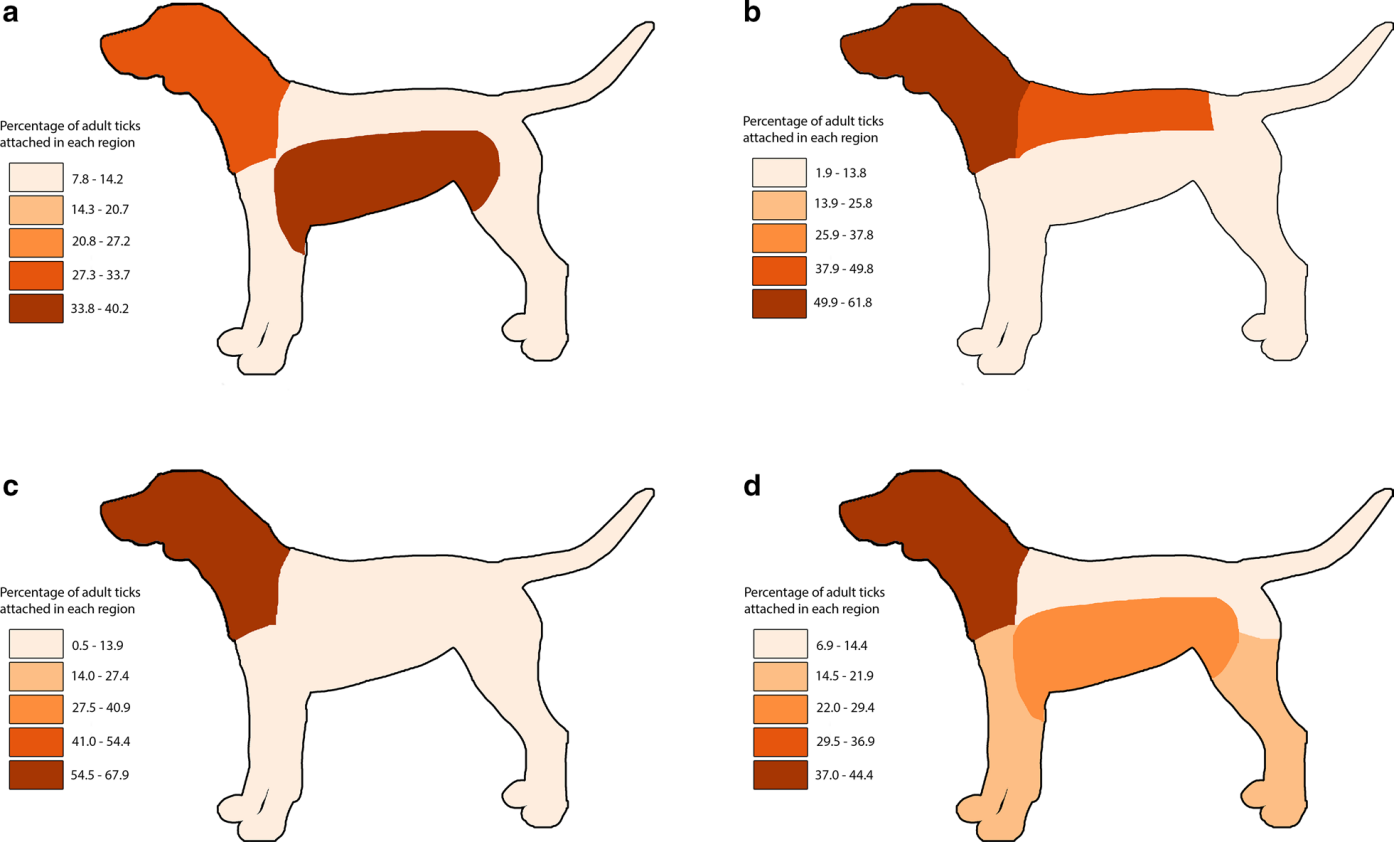
Distribution of attachment sites of adult ticks on dogs. **a** *Amblyomma americanum*. **b** *Dermacentor variabilis*. **c** *Ixodes scapularis*. **d** *Rhipicephalus sanguineus* (*sensu lato*)

Attachment site data from single-species and single-stage infestations of adult ticks were available for 169 dogs with *A. americanum*, 317 dogs with *I. scapularis*, 386 dogs with *D. variabilis* and 92 dogs with *R. sanguineus*. Reported tick attachment sites are summarized in Table 5 and Fig. 1. *Amblyomma americanum* was more commonly attached ventrally (*χ*^2^ = 27.6, *df* = 1, *P* < 0.0001); *D. variabilis, I. scapularis* and *R. sanguineus* were more commonly attached dorsally (*χ*^2^ = 43.1, *df* = 1, *P* < 0.0001; *χ*^2^ = 104.0, *df* = 1, *P* < 0.0001; *χ*^2^ = 6.4, *df* = 1, *P* = 0.0115, respectively). *Amblyomma americanum* was more commonly attached to the abdomen, axillary and inguinal region (*χ*^2^ = 85.25, *df* = 1, *P* < 0.0001); *Dermacentor variabilis* and *Ixodes scapularis* were more commonly attached to the head, ears and neck (*χ*^2^ = 15.43, *df* = 1, *P* = 0.0008; *χ*^2^ = 41.93, *df* = 1, *P* < 0.0001, respectively) and also to the back (*χ*^2^ = 14.64, *df* = 1, *P* = 0.0001; *χ*^2^ = 4.48, *df* = 1, *P* = 0.0342, respectively). *Rhipicephalus sanguineus* was more commonly attached to the head, ears and neck (*χ*^2^ = 16.97, *df* = 1, *P* = 0.00004), abdomen, axillary and inguinal regions (*χ*^2^ = 10.15, *df* = 1, *P* = 0.0014), as well as the legs and feet (*χ*^2^ = 29.76, *df* = 1, *P* < 0.0001).

**Table 5:** Number (percent) of adult ticks attached to different sites on dogs

Attachment site	*Amblyomma americanum*	*Dermacentor variabilis*	*Ixodes scapularis*	*Rhipicephalus sanguineus*
Ventral	156 (67.2)*	192 (35.8)	107 (25.2)	136 (42.9)
Dorsal	76 (32.8)	344 (64.2)*	317 (74.8)*	181 (57.1)*
Head, ears and neck	66 (28.4)	328 (61.2)*	287 (67.7)*	140 (44.2)*
Abdomen, axillary, inguinal	92 (39.7)*	64 (11.9)	41 (9.7)	77 (24.3)*
Legs and feet	28 (12.1)	37 (6.9)	17 (4.0)	53 (16.7)*
Back	28 (12.1)	97 (18.1)*	77 (18.2)*	25 (7.9)
Tail and perianal	18 (7.8)	10 (1.9)	2 (0.5)	22 (6.9)
Total	232	536	424	317

*Indicates a significant difference at α = 0.05

**Fig. 2:**
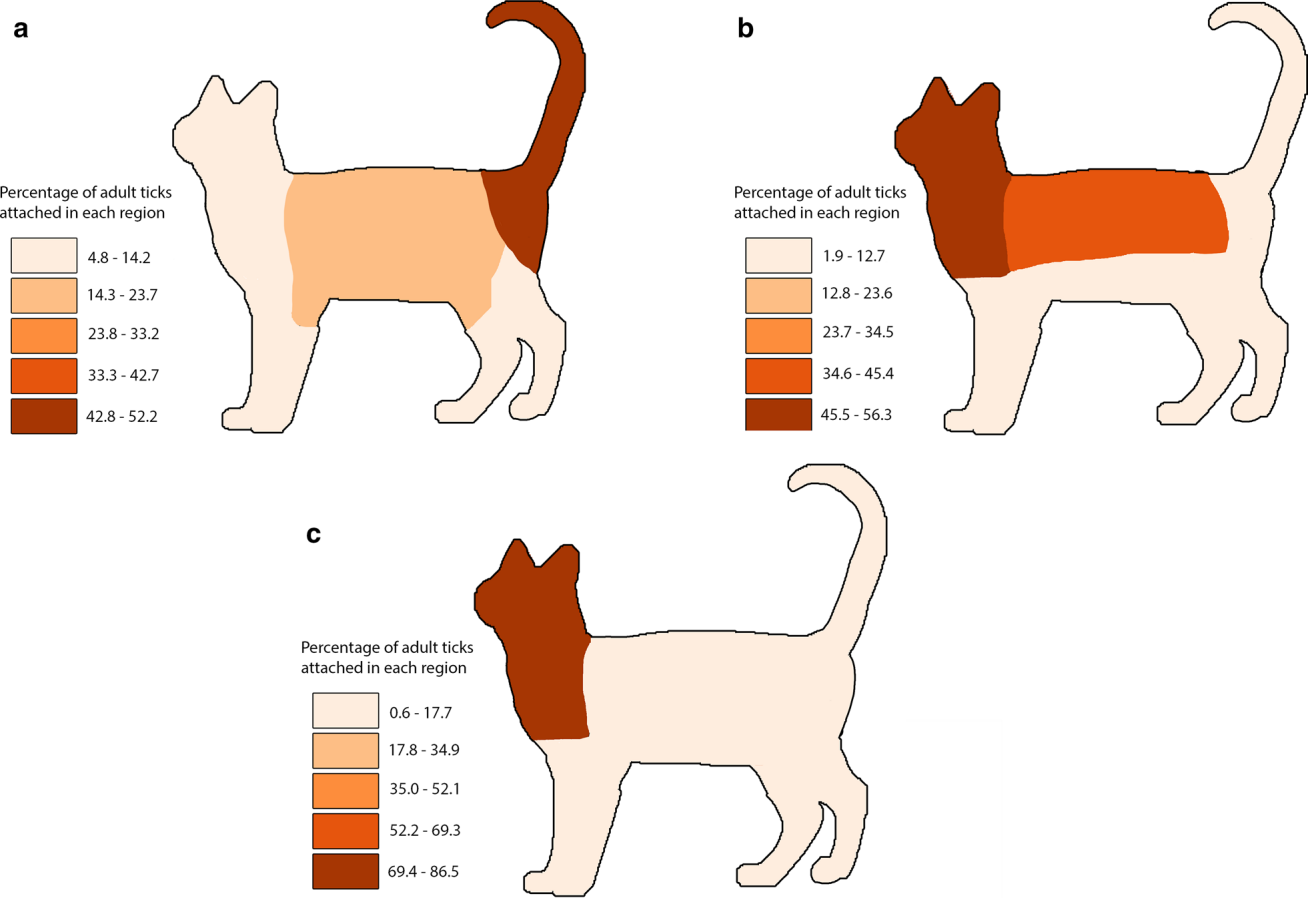
Distribution of attachment sites of adult ticks on cats. **a** *Amblyomma americanum*. **b** *Dermacentor variabilis*. **c** *Ixodes scapularis*

Attachment site data from single-species infestations were available for 33 cats with *A. americanum*, 116 cats with *I. scapularis* and 37 cats with *D. variabilis*. Reported tick attachment sites are summarized in Table 6 and Figure 2. *Amblyomma americanum* was more commonly attached ventrally (*χ*^2^ = 12.6, *df* = 1, *P* = 0.0004); *D. variabilis* and *I. scapularis* were more commonly attached dorsally (*χ*^2^ = 7.7, *df* = 1, *P* = 0.0055; *χ*^2^ = 14.9, *df* = 1, *P* = 0.0001). *Amblyomma americanum* was most commonly attached to the tail and perianal region (*χ*^2^ = 120.74, *df* = 1, *P* < 0.0001) and *I. scapularis* to the head, ears and neck (*χ*^2^ = 100.73, *df* = 1, *P* < 0.0001); *D. variabilis* did not have a statistically significant reported area of attachment (*χ*^2^ = 1.55, *df* = 1, *P* = 0.21).

**Table 6:** Number (percent) of adult ticks attached to different sites on cats

Attachment site	*Amblyomma americanum*	*Dermacentor variabilis*	*Ixodes scapularis*
Ventral	45 (72.6)*	16 (30.8)	62 (35.4)
Dorsal	17 (27.4)	36 (69.2)*	113 (64.6)*
Head, ears and neck	3 (4.8)	29 (55.8)	151 (86.3)*
Abdomen, axillary, inguinal	13 (21.0)	3 (5.8)	2 (1.1)
Legs and feet	5 (8.1)	2 (3.8)	4 (2.3)
Back	9 (14.5)	17 (32.7)	17 (9.7)
Tail and perianal	32 (51.6)*	1 (1.9)	1 (0.6)
Total	62	52	175

*Indicates a significant difference at α = 0.05

## Discussion

Our data confirm that tick infestations on dogs and cats in the USA are widespread. In the present study, ticks were identified from pets from a larger geographical area than has been reported in the USA [2, 4–14]. The primary tick species identified (*R. sanguineus*, *A. americanum*, *D. variabilis* and *I. scapularis*) constituted more than 95% of the ticks submitted from dogs, as was seen in earlier regional reports [6, 9]. Similarly, more than 80% of the ticks found on cats were *A. americanum*, *I. scapularis*, or *D. variabilis*, as has been described in previous surveys [2, 8–10]. Most pets with ticks had outdoor access, but a variety of tick species were submitted from a few dogs and cats that were reported to rarely or never go outside, suggesting ticks carried into the home on clothing or other pets may create a risk to indoor pets [2].

Less common tick species were also submitted from dogs and cats in the present study. Gulf Coast ticks, *A. maculatum*, were submitted from 98 dogs and 5 cats and have been previously reported from pets, but the geographical distribution appears to be expanding [6, 7, 9, 29]. Nymphs of *O. megnini* were submitted from the ear canals of 6 dogs and 13 cats. Although relatively uncommon, some spinose ear tick infestations in the present study were intense, with 26 nymphs from a single dog and 16 nymphs from a single cat, supporting the assertion that clinically relevant infestations with *O. megnini* occur in dogs and cats [1, 30, 31]. The Asian longhorned tick, *H. longicornis*, a species recently recognized in the USA [32], was submitted from 2 dogs and 1 cat. Longhorned ticks are commonly found on dogs and cats in other areas of the world where the species has long been present [33–35] and we expect to continue to identify this tick from pets in the USA in the future.

The present study also confirmed that immature stages of some common tick species readily infest dogs and cats. Larvae and nymphs constituted the majority of *A. americanum* and *R. sanguineus* submissions from dogs, corroborating on a national scale findings from a large, regional survey of ticks infesting dogs [6]. A majority of the *A. americanum* submitted from cats in the present study were also larvae or nymphs, an observation that has been described in earlier reports [2, 8]. Two cats harbored nymphs of *R. sanguineus* (*sensu lato*), a finding not previously reported in North America; adults of this species have been identified from cats in the USA and nymphs are reported from cats from other areas of the world [2, 9, 36]. Immature tick stages are important for pet health and may be overlooked due to their small size, an issue that can contribute to failing to recognize the complete tick risk faced by pets [2, 8, 9].

Host attachment site preferences also were evident among adult ticks in the present study. Adult *A. americanum* were more commonly attached ventrally and adult *D. variabilis*, *R. sanguineus* and *I. scapularis* were more commonly attached dorsally, as has been previously noted [2, 6]. In dogs in the present study, *D. variabilis* and *I. scapularis* were found more commonly attached to the head, ears, neck and back. In a survey of ticks removed from dogs in Europe, *I. ricinus* and *I. hexagonus* preferred the head and *D. reticulatus* the back [37, 38]. In dogs in the present study, *R*. *sanguineus* was more commonly attached to the head, ears and neck, as well as the legs and feet. This finding agrees with earlier reports in both the USA and Europe, where *R. sanguineus* was commonly found attached between the toes [6, 39].

Limitations with the present study include sample bias, incomplete data from all pets with ticks and the broad geography from which ticks were submitted. Even when outdoor access was indicated, we do not have precise habitat information for each pet. Cats also appear to be under-represented as hosts for ticks. Estimates suggest that cats outnumber dogs as pets in the USA [40], but less than 20% of submissions were from cats. However, we relied on ticks collected from veterinary visits and cats are not taken to the veterinarian as often as dogs [41]. Complete data on factors such as attachment site were not provided for every submission and attachment sites from co-infestations with multiple species or stages were not included in the analysis as the original location of each tick on the pet could not be determined. Omitting these co-infested pets from the attachment site analysis was necessary but limited the power of our results. Finally, phenology of ticks varies with geography, precluding complete analysis of seasonality in the present paper.

## Conclusions

This study revealed that a diverse array of ticks infest dogs and cats across the USA and throughout the year. Attachment site predilections were also confirmed, targeting key anatomic areas to examine when attempting to evaluate pets for active tick-infestation. This study also highlights the importance of broad-spectrum tick control in pets. Given the continued increase and geographical spread of tick populations in the USA [29] routine use of tick control is increasingly important for protecting pets from ticks. Surveillance of pets for ticks provides a valuable resource for understanding the tick risk faced by dogs, cats and people.

## Data Availability

Data supporting the conclusions of this article are included within the article. The summary datasets used and/or analyzed during the present study are available from the corresponding author upon reasonable request.

## Abbreviations

PCR: polymerase chain reaction
*16S* rRNA: 16S ribosomal RNA gene
*cox*1: cytochrome *c* oxidase subunit 1
ITS2: internal transcribed spacer 2
