# Correction to: Show us your ticks: a survey of ticks infesting dogs and cats across the USA

**DOI:** 10.1186/s13071-021-04599-4

**Published:** 2021-02-22

**Authors:** Meriam N. Saleh, Kellee D. Sundstrom, Kathryn T. Duncan, Michelle M. Ientile, Julia Jordy, Parna Ghosh, Susan E. Little

**Affiliations:** Department of Veterinary Pathobiology, Center for Veterinary Health Sciences, Oklahoma State University, Stillwater, OK 74078 USA

## Correction to: Parasites Vectors (2019) 12:595 10.1186/s13071-019-3847-3

Following publication of this article [1], the authors found an error in Table 4: the identity of the ticks from one cat, 32 larvae, had been incorrectly recorded as *Dermacentor variabilis*, while it is actually *Ixodes cookei*, as confirmed by re-examination of the original datasheet and tick specimens.

**Table 4:** Ticks collected from domestic cats in the USA by species, stage, and month of collection (corrected version)

Species	Stage	Total	Jan	Feb	Mar	Apr	May	Jun	Jul	Aug	Sep	Oct	Nov	Dec
*Amblyomma americanum*	F	93	0	0	2	6	45	33	6	0	1	0	0	0
	M	32	0	0	1	2	15	11	0	1	1	0	1	0
	N	92	0	0	2	9	27	20	5	22	6	1	0	0
	L	126	0	0	0	0	0	7	27	41	39	12	0	0
*Dermacentor variabilis*	F	48	0	0	1	1	20	13	7	1	1	0	4	0
	M	41	0	0	0	4	13	7	8	1	0	0	8	0
	N	1	0	0	0	0	0	0	0	1	0	0	0	0
	L	0	0	0	0	0	0	0	0	0	0	0	0	0
*Ixodes scapularis*	F	225	8	0	4	2	12	4	1	0	3	110	53	28
	M	41	2	0	0	0	1	2	0	0	0	28	5	3
	N	19	0	0	0	1	7	8	1	0	2	0	0	0
	L	2	0	0	0	0	0	2	0	0	0	0	0	0
Other	F	20	0	2	0	0	1	5	8	2	1	0	1	0
	M	4	1	0	0	0	0	0	3	0	0	0	0	0
	N	115	22	0	0	0	0	9	3	29	18	13	4	17
	L	32	0	0	0	0	0	0	0	0	32	0	0	0
Total		891	33	2	10	25	141	121	69	98	104	163	76	48

Other submitted ticks included *O. megnini*, *R. sanguineus* sensu lato, *A. maculatum*, *D. albipictus*, *I. affinis*, *I. angustus*, *I. cookei*, *I. pacificus*, *H. longicornis*, and *D. andersoni*

*F* female, *M* male, *N* nymph, *L* larva

Please note that this error did not affect the analysis or conclusions.

However, please be advised of the following corrections to the results: the number of different tick species found on cats (*n* = 13); the percentage and number of cats with *D. variabilis* (17.6% and 59/336); the number of cats with *I. cookei* (*n* = 1); the number and percentage of *D. variabilis* on cats (90/891 and 10.1%); the number of *I. cookei* on cats (*n* = 32); and the percentage of adult female *D. variabilis* in the study (679/1115 and 60.9%).

The table has now been corrected in the published article and is provided in this correction; likewise, the above corrections to the results have been made in the published article.

The authors apologize for this error and for any inconvenience it may have caused.
